# Does an Autoimmune Disorder Following Ovarian Cancer Diagnosis Affect Prognosis?

**DOI:** 10.3390/curroncol31080344

**Published:** 2024-08-13

**Authors:** Anaïs Fröhlich, JoEllen Welter, Isabell Witzel, Julia Voppichler, Mathias K. Fehr

**Affiliations:** 1 Department of Obstetrics and Gynecology, Spital Thurgau AG, 8501 Frauenfeld, Switzerland; joellen.welter@stgag.ch (J.W.); julia.voppichler@stgag.ch (J.V.); 2 Department of Gynecology, University Hospital Zürich, University of Zurich, 8091 Zurich, Switzerland; isabell.witzel@usz.ch

**Keywords:** ovarian cancer, autoimmune disorder, autoimmune disease, overall survival, prognosis

## Abstract

We investigated whether developing an autoimmune disorder (AID) following a high-grade epithelial ovarian cancer diagnosis improves overall survival. This retrospective study included data from women treated for high-grade serous, endometrioid, or transitional cell ovarian, fallopian tube, or peritoneal cancer FIGO stage III or IV at a Swiss cantonal gynecological cancer center (2008–2023). We used Kaplan–Meier estimates and the Cox proportional hazards model using time-varying covariates for the survival function estimation. In all, 9 of 128 patients developed an AID following a cancer diagnosis. The median time from cancer diagnosis to AID was 2 years (IQR 2–5). These women survived for a median of 3031 days (IQR 1765–3963) versus 972 days (IQR 568–1819) for those who did not develop an AID (*p* = 0.001). The median overall survival of nine women with a pre-existing AID was 1093 days (IQR 716–1705), similar to those who never had an AID. The multivariate analyses showed older age (*p* = 0.003, HR 1.04, 95% CI 1.013–1.064) was associated with a poorer prognosis, and developing an AID after a cancer diagnosis was associated with longer survival (*p* = 0.033, HR 0.113, 95% CI 0.015–0.837). Clinical manifestations of autoimmune disorders following ovarian cancer diagnoses were associated with better overall survival (8 versus 2.7 years), indicating an overactive immune response may improve cancer control.

## 1. Introduction

Ovarian cancer is the third most common gynecologic cancer worldwide [1]. It accounts for more deaths than any other malignancy of the female reproductive system [1,2,3], with approximately 75% of cases diagnosed at an advanced International Federation of Gynecology and Obstetrics (FIGO) stage [3,4]. Tumor debulking surgery and platinum–taxane-based chemotherapy remain the mainstay of ovarian cancer treatment [3,5]. Recent advances in maintenance therapy include vascular endothelial growth factor (VEGF) and poly(ADP-ribose) polymerase (PARP) inhibitors [3,6,7,8,9,10,11]. Factors known to affect prognosis include tumor stage at diagnosis, remaining tumor after debulking surgery, grading, histologic subtype, and age at disease onset [12,13]. Also, tumor-infiltrating lymphocytes have been shown to affect prognosis positively [14,15,16]. Unfortunately, dense tumor infiltration by lymphocytes occurs infrequently in ovarian cancer. Phase III studies with immune checkpoint inhibitors, which have been effective in treating malignant endometrial and cervical cancer, have not yet shown a significant benefit either as a monotherapy or when combined with chemotherapy [17,18,19,20,21,22,23,24,25]. Conversely, monogenic diseases such as small-cell carcinoma of the ovary, hypercalcemic type with loss-of-function of the SMARCA4 gene, have been treated successfully with checkpoint inhibitor therapy alone [26]. 

A recent study by Conrad et al. showed that approximately 10% of the population of the United Kingdom is affected by at least one autoimmune disorder. Of those, 63.9% are women. Given that more than 80 different types of autoimmune disorders have been identified and the incidence of autoimmune disorders continues to rise [27], further research into the interaction between an overactive immune response and a cancer prognosis is needed. 

The study’s primary objective was to compare the overall survival of women who developed an autoimmune disorder following a diagnosis of high-grade serous or endometrioid ovarian, fallopian tube, or peritoneal cancer FIGO stage III or IV with the survival of those who did not develop such a disorder after a cancer diagnosis. 

## 2. Materials and Methods

### 2.1. Study Design and Population

**Figure 1:**
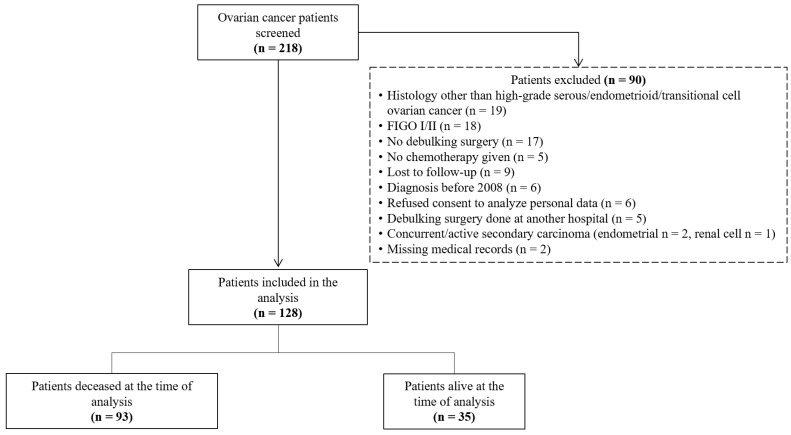
Flow diagram of ovarian cancer patient identification, screening, and inclusion in this retrospective analysis (n = 218).

This retrospective cohort study included women with high-grade serous, endometrioid, or transitional cell ovarian, tubal, or peritoneal cancers FIGO stage III or IV who underwent primary or interval debulking surgery and received neoadjuvant or adjuvant chemotherapy at two cantonal hospitals in Switzerland between January 2008 and December 2023. These two hospitals form a gynecological cancer center. The local ethics commission approved this study (2023-01696 EKOS 23/167), and a waiver was granted that allowed the use of data from 59 (46%) deceased patients without prior written consent. The exclusion of screened patients is shown in Figure 1.

### 2.2. Outcome Measures

The study’s primary objective was to compare the overall survival of women who developed an autoimmune disorder (including autoimmune diseases and autoimmune-related disorders) after a high-grade serous or endometrioid ovarian, fallopian tube, or peritoneal cancer FIGO stage III or IV diagnosis with the survival of those who did not develop an autoimmune system disorder after a cancer diagnosis. The variables assessed were age and body mass index at the time of cancer diagnosis, history of smoking (current or previous), race, other major comorbidities, BRCA 1/2 mutation, survival status, overall survival, and history of autoimmune-related disorder. The oncologic parameters were cancer type, histological subtype, nodal status, residual tumor burden, FIGO status, type of debulking surgery, adjuvant systemic therapy (including maintenance therapy), number of treatments for recurrence, and disease status at the time of analysis. 

Our definition of an autoimmune disorder was limited to diseases leading to systemic inflammation. Consequently, we excluded localized autoimmune disorders such as lichen sclerosus or thyroid autoimmune disease. We also included chronic lymphocytic leukemia causing a systemic autoimmune-associated condition, such as prurigo nodularis or immune thrombocytopenia. Overall survival was defined as the date from histological confirmation of cancer to the date of death from any cause. All patients still alive were censored using the last follow-up date. 

As a secondary analysis, we compared the overall survival of the following three groups: (1) no autoimmune disorder ever detected, (2) autoimmune disorder before ovarian cancer diagnosis, and (3) autoimmune disorder after ovarian cancer diagnosis.

### 2.3. Statistical Analysis

In addition to basic descriptive analyses, the data were compared by groups according to the status of autoimmune disorders. Continuous variables were summarized based on normality tests (Shapiro–Wilks), mean with standard deviation (±SD), or median (interquartile range IQR). Categorical variables were presented as frequency and percentages, and comparisons between groups were performed with Fisher’s exact test. Wilcoxon rank-sum tests were used for comparing continuous variables between two groups. Inferential statistics were carried out for the three-group comparisons using the one-way ANOVA, Kruskal–Wallis test, and Fisher’s exact test. Post hoc tests were performed with the Wilcoxon rank-sum and Fisher’s exact tests. In addition to the Kaplan–Meier estimator of survival function and the log-rank test for equality of survivor (non-parametric statistical test), we analyzed the association of time to death and the time-dependent covariate “autoimmune disorder after cancer diagnosis” using the Cox proportional hazards model by controlling for age at diagnosis, residual tumor burden, FIGO stage, and nodal status. To check the proportional hazards assumption, Schoenfeld residuals were correlated with time to test for independence between residuals and time. Log-linearity was checked with spline fits. As residual tumor burden clearly violated the proportional hazards assumption, a stratified Cox model was fitted with separate baseline hazards for patients with residual tumor burden R0, R1, and R2. In addition, we used the same model but excluded the nine patients with an autoimmune disorder at baseline (pre-existing disorder).

The alpha level to detect statistical significance when comparing the two main study groups (autoimmune-related disorder after cancer diagnosis and no autoimmune-related disorder after cancer diagnosis) was set at *p* < 0.05 for all tests. The Bonferroni correction was used to account for multiple-group comparisons for the secondary analyses using the three study groups. Therefore, *p*-values less than 0.0167 were considered statistically significant. All tests were two-sided, and we used the statistical software programs Stata (version 15, StataCorp, College Station, TX, USA) and R programming language (version 4.2.2, R Core Team, 2022) to conduct all the statistical analyses.

## 3. Results

The mean age at cancer diagnosis of the 128 study patients was 65.5 years (±10, range 32–83). The median body mass index was 25 kg/m^2^ (interquartile range [IQR] 22–28, range 17–47), 13% tested positive for BRCA 1/2 mutation (41% negative, 46% unknown/not tested), 10% had a history of smoking, 98% were white, and 69% had at least one major pre-existing comorbidity (hypertension being the most reported condition). Regarding oncological parameters, 93% had high-grade serous, 6% had high-grade endometrioid, and 1% had the high-grade transitional cell histological subtype. Residual tumor burdens were R0 in 43%, R1 in 47%, and R2 in 10% of patients. The nodal status distribution was 36% pN0 and 64% pN1. At the time of analysis, 11% of patients had no evidence of disease, 16% were under treatment, and 73% had died. The median number of recurrences was two (IQR 1–3), and the median survival time was 1020 days (IQR 586–1883, range 31–4989). Table 1 presents the demographic and clinical parameters grouped according to autoimmune disorder status.

All patients included underwent either primary (81%) or interval (19%) debulking surgery. Neoadjuvant or adjuvant first-line combination chemotherapy consisted of paclitaxel plus carboplatin. A total of 62% of patients (n = 79) had first-line maintenance therapy: 60 (47%) bevacizumab, 11 (9%) olaparib, and 8 (6%) niraparib.

Nine women (7%) developed an autoimmune disorder at a median of 2 years (IQR 2–5) after cancer diagnosis, and nine (7%) had a pre-existing autoimmune disorder at cancer diagnosis. The types of autoimmune disorders are shown in Table 2 and Table 3.

### 3.1. Primary Analysis (Two-Group Comparison)

**Figure 2:**
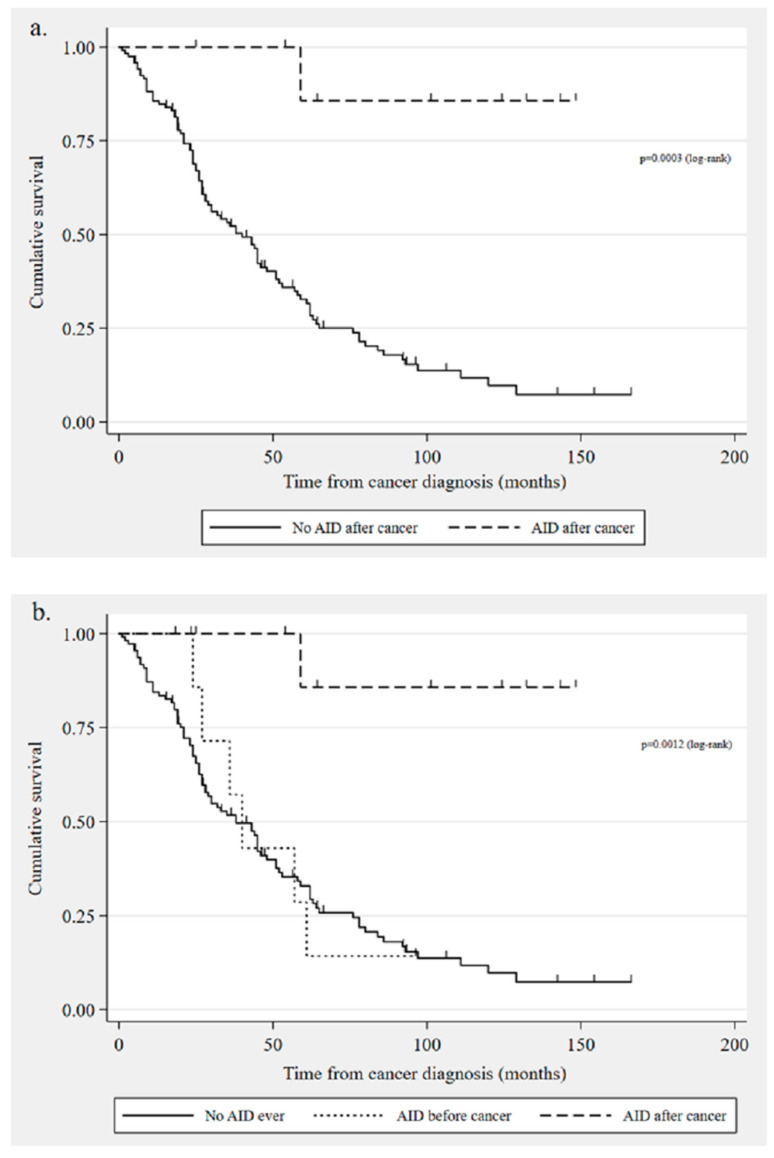
Kaplan–Meier survival curves: (**a**) overall survival in patients with autoimmune disorders developed after ovarian cancer diagnosis (n = 9) compared with those who did not develop a disorder after cancer (n = 119); (**b**) overall survival comparison: no autoimmune disorder diagnosis ever (n = 110), autoimmune disorder diagnosed before cancer (n = 9), and autoimmune disorder diagnosed after cancer (n = 9) [AID = autoimmune disorder].

When comparing the 119 women who did not develop an autoimmune disorder following a cancer diagnosis with the 9 women who did, we found a significantly longer median overall survival in the latter (3031 days (IQR 1765–3963, range 747–4443) versus 972 days (IQR 568–1819, range 31–4989, *p* = 0.001, see Figure 2a)). While 44% of the patients who developed an autoimmune disorder after a cancer diagnosis had no evidence of disease at the last follow-up, only 8% of the women in the other group were in remission (*p* = 0.001). Findings from the Cox proportional hazards analysis (124 cases included in the model) showed that developing an autoimmune disorder after a cancer diagnosis was associated with a better prognosis (*p* = 0.033, hazard ratio [HR] 0.113, 95% CI 0.015–0.837), whereas older age (*p* = 0.003, HR 1.04, 95% CI 1.013–1.064) was associated with poorer survival.

### 3.2. Secondary Analysis (Three-Group Comparison)

A comparison of the three groups showed that the median survival time was 1093 days (IQR 716–1705, range 538–2869) for the 9 women with pre-existing autoimmune disorder and 932 days (IQR 568–1846, range 31–4989) for the 110 patients who were never diagnosed with an autoimmune disorder in their lifetime. As mentioned in the previous section, the women with an onset of one of these disorders after a cancer diagnosis survived for a median of 3031 days (see Figure 2b). The difference in overall survival was significantly different between the group with an autoimmune disorder after cancer and the other two groups (*p* = 0.0011 no disorder ever and *p* = 0.0118 pre-existing disorder). However, there was no significant difference in survival times between the latter two groups (*p* = 0.7175). 

Using the Cox proportional hazards model (excluding patients with pre-existing autoimmune disorder), we found that age at diagnosis (*p* = 0.011, HR 1.034, 95% CI 1.008–1.061) was the only predictor variable associated with a poorer prognosis. However, developing an autoimmune disorder after a cancer diagnosis continued to be associated with longer survival time (*p* = 0.036, HR 0.116, 95% CI 0.016–0.865).

## 4. Discussion

The women who developed an autoimmune disorder following a high-grade ovarian, fallopian tube, or peritoneal cancer diagnosis FIGO stage III or IV lived significantly longer than those who did not develop such a disorder, with a three-fold increase in the median overall survival (8 versus 2.7 years). Forty-four percent of these patients had no evidence of disease at the last follow-up assessment. Older age at diagnosis was the only predictor variable negatively affecting overall survival. 

To the best of our knowledge, this is the first study investigating the impact of developing an autoimmune disorder following an ovarian cancer diagnosis on overall survival. Recently, the population-based Extreme study reported a poorer survival rate for ovarian cancer patients suffering from an autoimmune disease at the time of diagnosis [28]. Dedousis et al. investigated the presence of autoimmune disease in stage IV breast cancer prognosis [29]. They found that having an autoimmune disease prior to or after a diagnosis of metastatic breast cancer was associated with significantly longer overall and cancer-specific survival. However, in patients with stage I–III breast cancer, those with an autoimmune disease had a worse overall survival. 

In 2022, Wouters et al. conducted a systematic review showing a lower risk of ovarian cancer in patients with pre-existing multiple sclerosis (significant) or systemic lupus erythematosus (not significant) [30]. They suggested that their results may be due to increased numbers and altered function of regulatory T cells in the diseases mentioned above. For diabetes mellitus type 1, they found a higher incidence of ovarian cancer, and for the other eight autoimmune diseases, there was no significant effect at all. 

It is well established that a high density of tumor-infiltrating lymphocytes (especially CD8+) is associated with improved survival [14,15,16]. Consequently, immunotherapy has been the focus of several recently conducted clinical trials. In particular, immune checkpoint inhibitors such as programmed cell death protein 1 (PD-1) and programmed death-ligand 1 (PD-L1) pathway blockade have been examined. Despite their effectiveness in treating advanced malignant endometrial and cervical cancer, as well as non-gynecological tumors such as melanoma and non-small cell lung cancer (NSCLC) [22,24,31], ovarian cancer studies have failed to demonstrate a significant impact of checkpoint inhibitors [17,18,19,20,21,25].

On the other hand, several studies (mainly related to melanoma, NSCLC, renal cell carcinoma, or urothelial cell carcinoma) have assessed the association between immune-related adverse events and treatment efficacy in patients undergoing immune checkpoint inhibitor therapy [32,33,34]. They found a significant survival benefit among patients suffering from immune-related adverse events. A meta-analysis and systematic review by Du et al., which included 23 studies with a total of 22,749 patients suffering primarily from melanoma, NSCLC, or renal cell carcinoma, provided evidence of a significant outcome benefit among patients with cutaneous immune-related adverse events undergoing immune checkpoint therapy [32]. Overall survival (HR 0.61 [95% CI, 0.52–0.72]; *p* < 0.001), as well as progression-free survival (HR 0.52 [95% CI, 0.41–0.65]; *p* < 0.001), was improved. Similarly, Zhong et al. conducted a meta-analysis of 40 studies (mostly related to NSCLC and melanoma) that assessed other immune-related adverse events [33]. They also found a significantly longer overall survival (HR 0.49, 95% CI [0.42–0.58], *p* < 0.00001) in patients having side effects. The authors suggested a survival benefit in patients with endocrine, cutaneous, and gastrointestinal immune-related adverse events but not in pulmonary or hepatobiliary adverse events. Although our study did not assess the effects of immune checkpoint inhibitor therapy, our results also suggest that overstimulation of the immune system may improve cancer prognosis. 

We consider long-term follow-up assessments conducted in an outpatient clinic specializing in gynecologic oncology to be a notable advantage of this study. Consequently, survival data were available in most patients (93%). Unfortunately, survival data are often difficult to obtain [35], even in prospective phase III trials (personal communication steering committee of a phase III ovarian cancer trial). Another strength of this study was that our electronic medical records system is shared between the two participating cantonal hospitals, allowing the gynecologic oncologist to monitor the incidence of comorbidities at each follow-up visit. 

This study’s most important limitation is the small number of cases of pre-existing and post-diagnosis autoimmune disorders. Data had to be collected over a long period of time, during which treatment and diagnostic practices changed. For example, more recently diagnosed patients have benefitted from incorporating PARP inhibitors into clinical practice. Additionally, the autoimmune disorders were diverse, and medications were not reliably documented. Furthermore, we are limited to reporting deaths from all causes rather than cause-specific mortalities. Hence, our hypothesis-generating study needs confirmation with a larger cohort. 

Our study is the first to assess how developing an autoimmune disorder after an ovarian cancer diagnosis affects survival. The findings suggest that the antitumor activity of the immune system plays an important role in ovarian cancer, supporting the hypothesis that immunotherapy could be a promising treatment. Future research should elaborate upon how the immune system can be effectively stimulated without leading to major, often long-lasting side effects such as collagenosis. Translational research in this patient population is difficult since AIDs occur two to five years after the cancer diagnosis. Therefore, tumor samples taken at the time of cancer diagnosis may not predict tumor immunology at the onset of AID. Furthermore, most of the patients suffering from an AID had no residual tumor at the time of AID onset.

## 5. Conclusions

The onset of an autoimmune disorder following an ovarian cancer diagnosis was associated with better overall survival. These findings support the theory that immune system stimulation may strikingly improve survival for high-grade epithelial ovarian cancer patients.

## Figures and Tables

**Table 1:** Demographic characteristics and clinical parameters according to autoimmune disorder status.

Parameters	No AID Ever		AID before Cancer Diagnosis		AID after Cancer Diagnosis		*p*-Value **
Total (n = 128)	n = 110		n = 9		n = 9		--
Age at cancer diagnosis (mean, standard deviation)	65.5	±10	62.8	±11	65	±6	0.246
Body mass index-kg/m^2^ (median, IQR)	25	22–28	23	23–26	27	23–28	0.641
Race (white) *	108	98%	9	100%	9	100%	0.999
Smoker (yes)	11	10%	1	11%	1	11%	0.999
BRCA mutation (yes)	12	11%	2	22%	2	22%	0.048
Histologic subtype							0.7556
Serous	102	93%	8	89%	9	100%	
Endometrioid	7	6%	1	11%	0	--	
Transitional cell	1	1%	0	--	0	--	
No residual tumor (R0)	44	40%	4	44%	6	67%	0.321
Nodal status *							0.413
pN0	36	34%	4	44%	5	56%	
pN1	70	66%	5	56%	4	44%	
FIGO							0.76
III	80	74%	6	67%	6	67%	
IV	30	26%	3	33%	3	33%	
Cycles of primary chemotherapy ^†^ (median, range)	6	1–6	6	5–6	6	6–6	0.837
First-line maintenance therapy (yes)	65	59%	7	78%	7	78%	0.324
Treatments for recurrence (median, range)	2	1–9	2	1–3	2.5	2–3	0.7018
Survival time in days (median, IQR)	932	567–1846	1093	716–1705	3031	1765–3963	0.0042
No evidence of disease at last follow-up (yes)	9	8%	1	11%	4	44%	0.012

* Missing data; ** Bonferroni correction (*p* < 0.0167); AID = autoimmune disorder; ^†^ paclitaxel/carboplatin.

**Table 2:** Demographic and clinical characteristics of patients with autoimmune disorders after cancer diagnosis (n = 9).

Case	Age (Years)	FIGO Stage	Residual Tumor	BRCA 1/2 Mutation	Maintenance Therapy	Autoimmune Disorder	Time to Disease *	Autoimmune Disorder Therapy	Recurrences (Number)	Survival Status	Survival Time (Days)
1	72	III	R1	Unknown	Bevacizumab	Polymyalgia rheumatica	5 years	Prednisone	0	Alive	3716
2	64	IV	R0	Negative	Bevacizumab, niraparib	Rheumatoid arthritis	4 years	Etanercept, methotrexate	3	Alive	4290
3	67	III	R2	Negative	Bevacizumab	Granulomatosis with polyangiitis (Wegener’s disease)	2 years	Methotrexate	2	Died	1765
4	63	IV	R0	Unknown	--	Chronic lymphocytic leukemia with prurigo nodularis	6 years	Corticosteroid (local)	0	Alive	4443
5	57	III	R0	Negative	Bevacizumab, niraparib	Rheumatoid arthritis	2 years	Prednisone, methotrexate	2	Alive	1627
6	67	IV	R1	Positive	Olaparib	Systemic lupus erythematosus	5 years	Corticosteroid (local)	3	Alive	3031
7	76	III	R0	Negative	Bevacizumab	Rheumatoid arthritis	2 years	Prednisone	0	Alive	1930
8	58	III	R0	Negative	--	Dermatomyositis	1 year	Prednisone, methotrexate, hydroxychloroquine, mycophenolic acid, rituximab, corticosteroid (local)	0	Alive	3963
9	60	III	R0	Positive	Olaparib	Leukocytoclastic vasculitis	1.5 years	Prednisone, enoxaparin	0	Alive	747

* Time from cancer diagnosis to detection of autoimmune disorder.

**Table 3:** Demographic and clinical characteristics of patients with autoimmune disorder(s) before cancer diagnosis (n = 9).

Case	Age (Years)	FIGO Stage	Residual Tumor	BRCA 1/2 Mutation	Maintenance Therapy	Autoimmune Disorder	Time to Disease *	Autoimmune Disorder Therapy	Recurrences (Number)	Survival Status	Survival Time (Days)
1	76	III	R2	Negative	Bevacizumab	Rheumatoid arthritis	Unknown ^†^	Methotrexate, deflazacort	1	Died	806
2	69	IV	R2	Negative	Bevacizumab, olaparib	Polymyalgia rheumatica	11 years	Prednisone	2	Alive	676
3	65	IV	R2	Negative	Bevacizumab, olaparib	Rheumatoid arthritis	2 months	Unknown ^†^	3	Died	716
4	51	III	R2	Positive	Bevacizumab, olaparib	Systemic lupus erythematosus	2 years	Hydroxychoroquine, prednisone	3	Died	1705
5	75	IV	R0	Unknown	Bevacizumab	Chronic lymphocytic leukemia with immune thrombocytopenia	15 years	Rituximab, bendamustine, prednisone	1	Died	1093
6	67	III	R0	Negative	Bevacizumab	Ankylosing spondylitis (Bechterew’s disease)	Unknown ^†^	Local steroids	1	Died	1206
7	61	III	R0	Negative	--	Ulcerative colitis	7 years	Mesalazine	2	Alive	2869
8	59	III	R0	Negative	Bevacizumab, olaparib	Mixed connective tissue disease	3 years	Hydroxychloroquine, prednisone, sulfasalazine, methotrexate, cyclophosphamide, rituximab	2	Alive	538
9	42	III	R2	Positive	--	Autoimmune diabetes mellitus (Type 1)	3 years	Insulin detemir, insulin aspart	3	Died	1819

^†^ Missing from medical records; * time from cancer diagnosis to detection of autoimmune disorder.

## Data Availability

The raw data supporting the conclusions of this article will be made available by the authors on request.
